# Supplementary figures and images for: Variability in intrinsic promoter strength underlies the temporal hierarchy of the Caulobacter SOS response induction
Source: PLoS Biol. 2025 Dec 4;23(12):e3003557. doi: 10.1371/journal.pbio.3003557 (PMC12700426; doi:10.1371/journal.pbio.3003557)

Figure S1

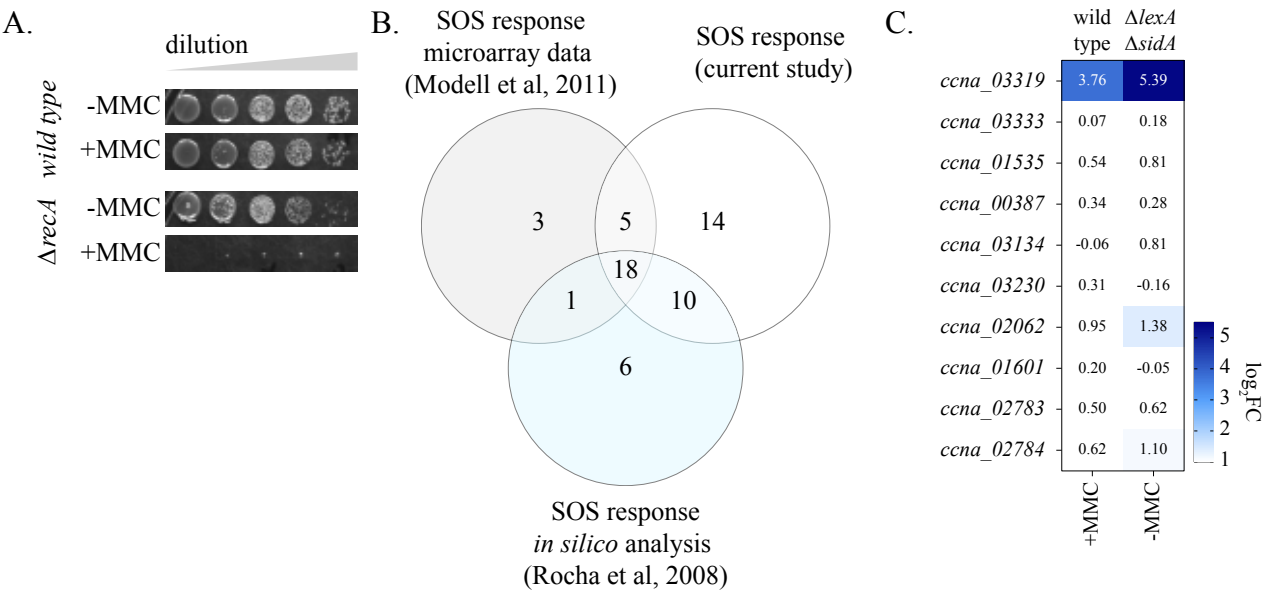

Supplement: S1 Fig — (A) Survival assay of wild type and ΔrecA cells in the presence and absence of MMC (0.25 μg/mL). Representative image shown from three biological replicates. (B) Venn diagram comparing SOS response genes identified in this study with the in silico analysis from Rocha and colleagues, 2008 [27] and microarray at 40 min post MMC damage from Modell and colleagues, [28]. Number of genes in each category are indicated. The underlying data are available in S1 Data. (C) Heat map for log2FC values of genes previously identified as Caulobacter SOS response genes which do not fulfill criteria put forth in this study. Log2FC values for an SOS-induced gene (ccna_03319) in wild type cells exposed to MMC damage for 40 min and for ΔlexAΔsidA cells in the absence of damage are shown for comparison. (PDF) [file pbio.3003557.s001.pdf]

Figure S2

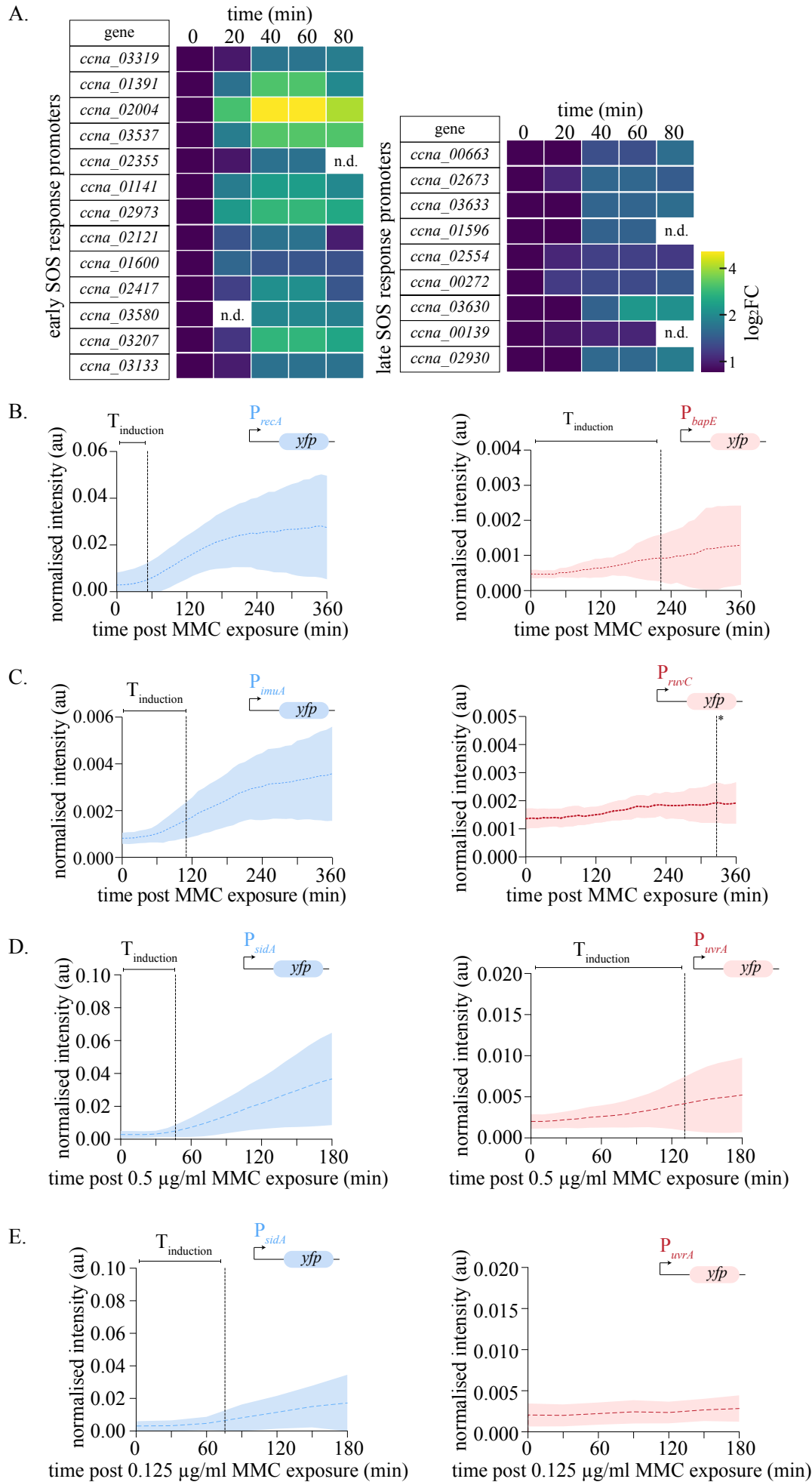

Supplement: S2 Fig — (A) Heat map showing gene expression changes at 20, 40, 60, and 80 min post MMC damage. Data are replotted from microarray analysis in [28]. Genes are marked as early or late based on their time to induction. The underlying data are available in S1 Data. (B) Fluorescence intensity normalized to cell area for PrecAyfp (n = 25) [left] and PbapEyfp (n = 20) [right] cells over 6 hours of 0.25 μg/mL MMC exposure. Images were taken every 10 min and intensity over time traces are shown. Dashed line indicates the mean and the shaded region indicates the standard deviation. Time to induction (Tind - time to doubling of mean initial fluorescence intensity) is indicated on the graph. The underlying data are available in S1 Data. (C) As (B) for PimuAyfp (n = 25) [left] and PruvCyfp (n = 20) [right] cells over 6 hours of 0.25 μg/mL MMC exposure. Fluorescence intensity for PruvCyfp increases over time but does not cross the threshold set for time to induction within the imaging period. Time to normalized intensity maxima for PruvCyfp is indicated with an asterix. The underlying data are available in S1 Data. (D) As (B) for PsidAyfp [left] and PuvrAyfp [right] cells over 3 hours of MMC exposure (0.5 μg/mL, n = 25). The underlying data are available in S1 Data. (E) As (D) for cells treated with 0.125 μg/mL MMC, except for an imaging interval of 30 min (n = 20). At these low doses induction of yfp from the uvrA promoter is not detected. The underlying data are available in S1 Data. (PDF) [file pbio.3003557.s002.pdf]

Figure S4

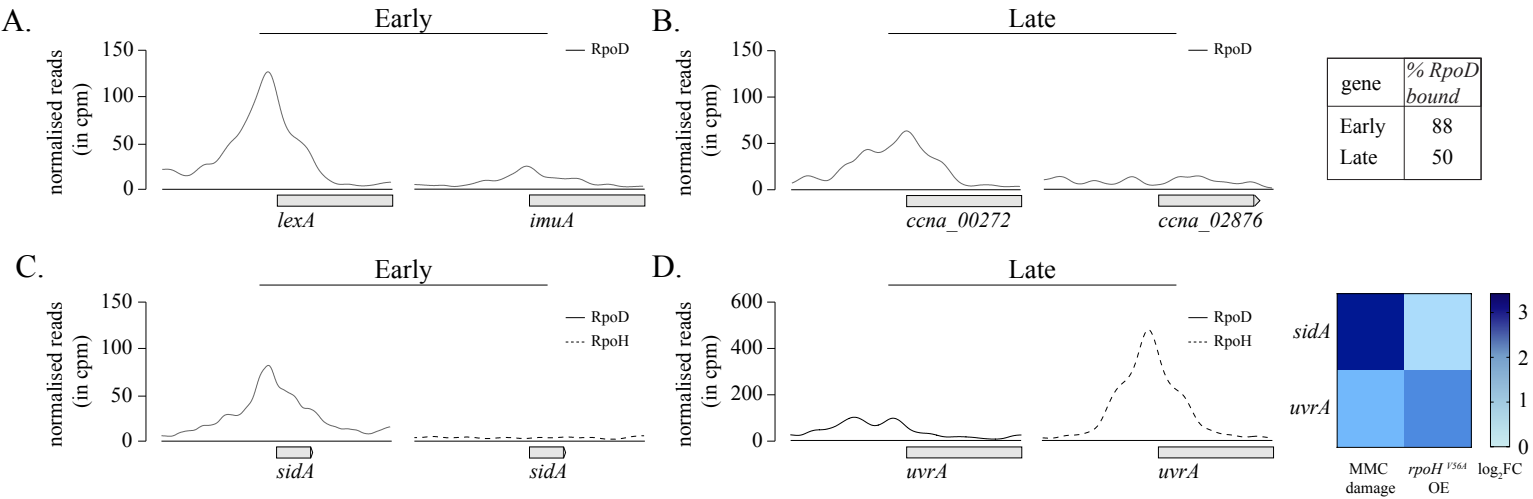

Supplement: S4 Fig — (A, B) ChIP-seq profiles for RpoD enrichment 500 bp upstream and downstream of the CDS for early [left] and late [right] SOS response genes. % SOS response genes bound by RpoD are indicated adjacent to the ChIP profiles. The underlying data are available in S1 Data. ChIP-seq data were obtained from the GEO database (GSE73925). (C, D) ChIP-seq profiles for RpoD and RpoH enrichment 500 bp upstream and downstream of the CDS of early (sidA) and late (uvrA) SOS response genes. The underlying data are available in S1 Data. ChIP-seq data were obtained from GEO database (GSE73925) [38]. Heat map of log2FC values from RNA-seq experiments for Caulobacter SOS response genes upon rpoHV65A over-expression is indicated adjacent to the ChIP profiles. RNA-seq data were obtained from GEO database (GSE102372) [35]. For comparison, log2FC values for the same genes under 40 min of MMC damage (0.25 μg/mL) are shown. (PDF) [file pbio.3003557.s004.pdf]
