# Supplementary material for: Variability in intrinsic promoter strength underlies the temporal hierarchy of the Caulobacter SOS response induction
Source: PLoS Biol. 2025 Dec 4;23(12):e3003557. doi: 10.1371/journal.pbio.3003557 (PMC12700426; doi:10.1371/journal.pbio.3003557)

Figure S3

A.

gene	LexA box	length
ccna_03319	GTTCCACTTTTGTTC	15
ccna_01391	GTTCTTGTTATGTTC	15
ccna_02004.1	GTTCACAAGATGTTC	15
ccna_02004.2	TTTCTTGTTTTGTTC	15
ccna_03118	GTTCCTTTTTTGTTC	15
ccna_03537	GTTCCTGAATTGTTC	15
ccna_02355	GTTCTTGTTATGTTC	15
ccna_01141	GTTCGCAAGATGTTC	15
ccna_02973	GTTCTGACTATGTTC	15
ccna_00386	GTTCCTGAAAAGTTC	15
ccna_00663	GTTCGCGTTATGTTC	15
ccna_01979	GTTCTCCTGGTGTTC	15
ccna_03826	GTTCCGCTTTCGTTC	15
ccna_02121	GTTCCCTTTCTGTTTC	15
ccna_01600	GTTCTTGATATGTTC	15
ccna_02417	GTTCTTGATTTGTTC	15
ccna_03580	GTTCCAGCTTTGTTC	15
ccna_03207	GTTCCCCTCTTGTTC	15
ccna_01106	GTTCTCGGCTTGTTC	15
ccna_03133	GTTCCTATAATGTTC	15
ccna_02673	GTTCGCATCTTGTTC	15
ccna_03346	GTTCATCATGTGTTC	15
ccna_03633	GTTCATGTATTGTTC	15
ccna_02876	GTTCTCTATTCGTTC	15
ccna_01596	GTGCTACATATGTTC	15
ccna_02554	GTTCTTGTTTCGTCC	15
ccna_00272	GTTCATGCTCCGTTC	15
ccna_03231	TTTCCGGATTTGTTC	15
ccna_02930	GTTCCTGTATTGTTC	15
ccna_03466	GTTCTCGTATTGTTC	15
ccna_03630	GTTCGCATTATGTTC	15
ccna_00139	GTTCGTTTTTCGTTC	15

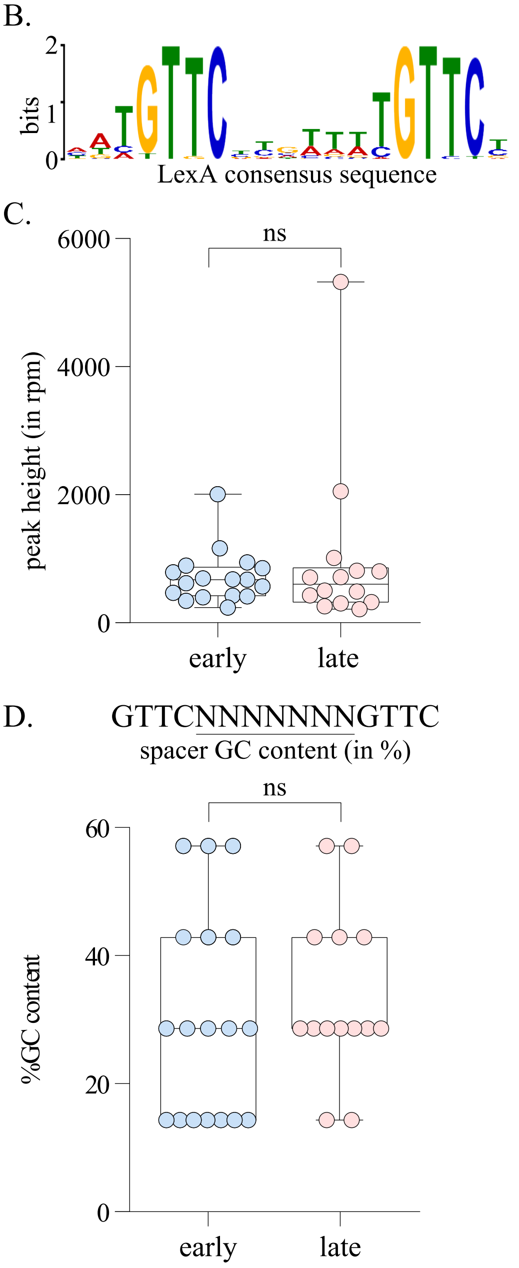

Supplement: S3 Fig — (A) List of LexA box sequence and their box length (in bp) for promoters of all Caulobacter SOS response genes analyzed in present study. (B) Binding consensus motif for LexA, based upon the promoters of the Caulobacter SOS response genes. (C) Box and scatter plot indicating peak height derived from LexA ChIP-seq data for early (blue) and late (red) SOS response genes. Mann–Whitney test, n.s.—not significant. The underlying data are available in S1 Data. (D) Box and scatter plot indicating %GC content of the LexA box spacer for the CDS of early (blue) and late (red) SOS response genes. Mann–Whitney test, n.s. —not significant. The underlying data are available in S1 Data. (PDF) [file pbio.3003557.s003.pdf]
