# Supplementary material for: Variability in intrinsic promoter strength underlies the temporal hierarchy of the Caulobacter SOS response induction
Source: PLoS Biol. 2025 Dec 4;23(12):e3003557. doi: 10.1371/journal.pbio.3003557 (PMC12700426; doi:10.1371/journal.pbio.3003557)
Supplement: S1 Table — (DOCX) [file pbio.3003557.s005.docx]

**Table S1: Strains used in present study**

| **Strain** | **Genotype** | **Strain construction** |
| --- | --- | --- |
| CB15N | NA1000 | NA |
| NABC2 | CB15N*; ∆recA* | [1] |
| NABC520 | CB15N; ΔlexA::tetR ; ΔsidA::markerless | [2] |
| NABC454 | CB15N*; pMT687-P_sidA_-YFP::kanR* | [3] |
| NABC891 | CB15N; *pMT687-PuvrA-YFP::kanR* | CB15N was transformed with pNABC881 plasmid |
| NABC969 | CB15N; *ΔlexA::tetR ; ΔsidA::markerless; pMT687-PsidA-YFP::kanßR* | NABC520 was transformed with pNABC498 plasmid |
| NABC970 | CB15N; *ΔlexA::tetR ; ΔsidA::markerless; pMT687-PuvrA-YFP::kanR* | NABC520 was transformed with pNABC881 plasmid |
| NABC1018 | CB15N*; pMT687-P_recA_-YFP::kanR* | CB15N was transformed with pNABC1012 plasmid |
| NABC1019 | CB15N*; pMT687-P_bapE_-YFP::kanR* | CB15N was transformed with pNABC1013 plasmid |
| NABC1020 | CB15N*; pMT687-P_imuA_-YFP::kanR* | CB15N was transformed with pNABC1014 plasmid |
| NABC1021 | CB15N*; pMT687-P_ruvC_-YFP::kanR* | CB15N was transformed with pNABC1015 plasmid |
| NABC1023 | CB15N*; pMT687-P_ccna_02355_-YFP::kanR* | CB15N was transformed with pNABC1017 plasmid |
| NABC1022 | CB15N*; pMT687-P_ccna_01391_-YFP::kanR* | CB15N was transformed with pNABC1016 plasmid |

**References:**

1. Modell JW, Kambara TK, Perchuk BS, Laub MT. A DNA Damage-Induced, SOS-Independent Checkpoint Regulates Cell Division in Caulobacter crescentus. PLoS Biology. 2014;12: e1001977. doi:10.1371/journal.pbio.1001977

2. Modell JW, Hopkins AC, Laub MT. A DNA damage checkpoint in Caulobacter crescentus inhibits cell division through a direct interaction with FtsW. Genes Dev. 2011;25: 1328–1343. doi:10.1101/gad.2038911

3. Chimthanawala A, Parmar JJ, Kumar S, Iyer KS, Rao M, Badrinarayanan A. SMC protein RecN drives RecA filament translocation for in vivo homology search. Proceedings of the National Academy of Sciences. 2022;119: e2209304119. doi:10.1073/pnas.2209304119
