# Supplementary material for: Variability in intrinsic promoter strength underlies the temporal hierarchy of the Caulobacter SOS response induction
Source: PLoS Biol. 2025 Dec 4;23(12):e3003557. doi: 10.1371/journal.pbio.3003557 (PMC12700426; doi:10.1371/journal.pbio.3003557)
Supplement: S2 Table — (DOCX) [file pbio.3003557.s006.docx]

**Table S2: Plasmids used in present study**

| **Plasmid** | **Construction details** | **Antibiotic** |
| --- | --- | --- |
| pRXMCS-2 | [1] | Kanamycin |
| pNABC498 | [2] | Kanamycin |
| pNABC881 | pNABC498 vector was amplified using AB_oligo_566 and AC_oligo_354 and P*_uvrA_* fragment was amplified from *Caulobacter* genomic DNA (gDNA) using AK_oligo_349 and AK_oligo_350. The vector and insert fragments were assembled with Gibson assembly. | Kanamycin |
| pNABC1012 | pNABC498 vector was amplified using AB_oligo_566 and AC_oligo_354 and P*_recA_* fragment was amplified from *Caulobacter* genomic DNA (gDNA) using AK_oligo_380 and AK_oligo_381. The vector and insert fragments were assembled with Gibson assembly. | Kanamycin |
| pNABC1013 | pNABC498 vector was amplified using AB_oligo_566 and AC_oligo_354 and P*_bapE_* fragment was amplified from *Caulobacter* genomic DNA (gDNA) using AK_oligo_376 and AK_oligo_377. The vector and insert fragments were assembled with Gibson assembly. | Kanamycin |
| pNABC1014 | pNABC498 vector was amplified using AB_oligo_566 and AC_oligo_354 and P*_imuA_* fragment was amplified from *Caulobacter* genomic DNA (gDNA) using AK_oligo_378 and AK_oligo_379. The vector and insert fragments were assembled with Gibson assembly. | Kanamycin |
| pNABC1015 | pNABC498 vector was amplified using AB_oligo_566 and AC_oligo_354 and P*_ruvC_* fragment was amplified from *Caulobacter* genomic DNA (gDNA) using AK_oligo_382 and AK_oligo_383. The vector and insert fragments were assembled with Gibson assembly. | Kanamycin |
| pNABC1017 | pNABC498 vector was amplified using AB_oligo_566 and AC_oligo_354 and P*_ccna_01391_* fragment was amplified from *Caulobacter* genomic DNA (gDNA) using AK_oligo_372 and AK_oligo_373. The vector and insert fragments were assembled with Gibson assembly. |  |
| pNABC1016 | pNABC498 vector was amplified using AB_oligo_566 and AC_oligo_354 and P*_ccna_02355_* fragment was amplified from *Caulobacter* genomic DNA (gDNA) using AK_oligo_374 and AK_oligo_375. The vector and insert fragments were assembled with Gibson assembly. |  |

**References:**

1. Thanbichler M, Iniesta AA, Shapiro L. A comprehensive set of plasmids for vanillate- and xylose-inducible gene expression in *Caulobacter crescentus*. Nucleic Acids Res. 2007;35: e137. doi:10.1093/nar/gkm818

2. Chimthanawala A, Parmar JJ, Kumar S, Iyer KS, Rao M, Badrinarayanan A. SMC protein RecN drives RecA filament translocation for in vivo homology search. Proceedings of the National Academy of Sciences. 2022;119: e2209304119. doi:10.1073/pnas.2209304119
