# Supplementary material for: Variability in intrinsic promoter strength underlies the temporal hierarchy of the Caulobacter SOS response induction
Source: PLoS Biol. 2025 Dec 4;23(12):e3003557. doi: 10.1371/journal.pbio.3003557 (PMC12700426; doi:10.1371/journal.pbio.3003557)
Supplement: S3 Table — (DOCX) [file pbio.3003557.s007.docx]

**Table S3: Oligos used in present study**

| **Oligo** | **Sequence** |
| --- | --- |
| AB_oligo_566 | ATGGTGAGCAAGGGCGAGGAG |
| AC_oligo_354 | ATGGCGAATGGCGCCGCGCTGATGTCCGGCGGTG |
| AK_oligo_349 | CACCGCCGGACATCAGCGCGGCGCCATTCGCCATAGAGCACGTTCGGAGGCGTAAA |
| AK_oligo_350 | GGTGAACAGCTCCTCGCCCTTGCTCACCATTTTCAGATTGTGCTCACGGGCACC |
| AK_oligo_372 | gccggacatcagcgcggcgccattcgccatcggagctttcctgatgcaagcg |
| AK_oligo_373 | ggtgaacagctcctcgcccttgctcaccatagactcgaagcgtcccgtccgg |
| AK_oligo_374 | gccggacatcagcgcggcgccattcgccatccggcgtcgatctggccag |
| AK_oligo_375 | ggtgaacagctcctcgcccttgctcaccatgcacaggagagcgtcccgca |
| AK_oligo_376 | gccggacatcagcgcggcgccattcgccatacaattggcccgacggcgtt |
| AK_oligo_377 | ggtgaacagctcctcgcccttgctcaccatcgtgatcagatcggcacggact |
| AK_oligo_378 | gccggacatcagcgcggcgccattcgccatccacgctctcgagccccg |
| AK_oligo_379 | ggtgaacagctcctcgcccttgctcaccatcgcggcgatccggcctct |
| AK_oligo_380 | gccggacatcagcgcggcgccattcgccatgcggcgcggactaaaccca |
| AK_oligo_381 | ggtgaacagctcctcgcccttgctcaccattgttgatccgccttctattccatgattccg |
| AK_oligo_382 | gccggacatcagcgcggcgccattcgccatctcggccgtctgcgcgttcg |
| AK_oligo_383 | ggtgaacagctcctcgcccttgctcaccatgccgggatcaaggccgaggatc |
