# Supplementary material for: Variability in intrinsic promoter strength underlies the temporal hierarchy of the Caulobacter SOS response induction
Source: PLoS Biol. 2025 Dec 4;23(12):e3003557. doi: 10.1371/journal.pbio.3003557 (PMC12700426; doi:10.1371/journal.pbio.3003557)
Supplement: S5 Table — (DOCX) [file pbio.3003557.s009.docx]

**Table S5: List of *Caulobacter* SOS response genes and their gene annotations**

| gene | protein name | *gene name* |
| --- | --- | --- |
| *ccna_03319* | ImuA | *imuA* |
| *ccna_03318* | ImuB | *imuB* |
| *ccna_03317* | Error-prone DNA polymerase | *dnaE2* |
| *ccna_01391* | Radical SAM superfamily protein | *ccna_01391* |
| *ccna_02004* | Cell division inhibitor SidA | *sidA* |
| *ccna_02003* | DNA polymerase III subunit alpha | *dnaE* |
| *ccna_03118* | Uncharacterized protein | *ccna_03118* |
| *ccna_03537* | SOS-regulated glyoxalase family protein | *mmcA* |
| *ccna_02355* | Endonuclease III | *ccna_02355* |
| *ccna_01141* | Protein RecA | *recA* |
| *ccna_02973* | Antitoxin protein relB-3 | *relB-3* |
| *ccna_02974* | Toxin protein relE3 | *relE3* |
| *ccna_00386* | DNA-3-methyladenine glycosylase | *ccna_00386* |
| *ccna_00663* | Bacterial apoptosis endonuclease BapE | *bapE* |
| *ccna_00662* | Uncharacterized protein | *ccna_00662* |
| *ccna_01979* | LexA repressor | *lexA* |
| *ccna_03826* | Pyocin large subunit family protein | *ccna_03826* |
| *ccna_03825* | DUF5076 domain-containing protein | *ccna_03825* |
| *ccna_02122* | DNA ligase-associated metallophosphoesterase | *ccna_02122* |
| *ccna_02121* | ATP-dependent helicase | *ccna_02121* |
| *ccna_01600* | Methane oxygenase, PmoA family | *ccna_01600* |
| *ccna_02418* | Type-4 uracil-DNA glycosylase | *ccna_02418* |
| *ccna_02417* | Biotin synthase related domain containing protein | *ccna_02417* |
| *ccna_03580* | SOS-induced DUF1052 family protein | *mmcB* |
| *ccna_03207* | DUF72 domain-containing protein | *ccna_03207* |
| *ccna_01106* | Antitoxin protein parD2 | *parD2* |
| *ccna_01107* | Toxin protein parE2 | *parE2* |
| *ccna_03133* | Toxin protein higB | *higB* |
| *ccna_03132* | Transcriptional regulator, antitoxin protein higA | *higA* |
| *ccna_03131* | LytR/AlgR-family transcriptional regulator | *ccna_03131* |
| *ccna_02673* | UvrABC system protein A | *uvrA* |
| *ccna_03346* | Crossover junction endodeoxyribonuclease RuvC | *ruvC* |
| *ccna_03345* | Holliday junction branch migration complex subunit RuvA | *ruvA* |
| *ccna_03344* | Holliday junction branch migration complex subunit RuvB | *ruvB* |
| *ccna_03343* | FtsW related protein | *ccna_03343* |
| *ccna_03342* | Short-chain acyl-CoA hydrolase | *ccna_03342* |
| *ccna_03633* | GIY-YIG domain protein | *ccna_03633* |
| *ccna_02876* | Very short patch repair (Vsr) endonuclease | *ccna_02876* |
| *ccna_01596* | DNA 3'-5' helicase | *uvrD* |
| *ccna_01595* | Conjugal transfer protein TrbI | *ccna_01595* |
| *ccna_02554* | DUF1640 domain-containing protein | *ccna_02554* |
| *ccna_00272* | DNA recombination protein RmuC homolog | *ccna_00272* |
| *ccna_03231* | Anti-toxin protein relB-4 | *relB-4* |
| *ccna_02930* | Very short patch repair (Vsr) endonuclease | *ccna_02930* |
| *ccna_03466* | ORF6N domain protein | *ccna_03466* |
| *ccna_03630* | Antitoxin SocA | *socA* |
| *ccna_00139* | Magnesium chelatase subunit ChlI-like ATPase | *chlI* |
