# Supplementary material for: Variability in intrinsic promoter strength underlies the temporal hierarchy of the Caulobacter SOS response induction
Source: PLoS Biol. 2025 Dec 4;23(12):e3003557. doi: 10.1371/journal.pbio.3003557 (PMC12700426; doi:10.1371/journal.pbio.3003557)
Supplement: S7 Table — (DOCX) [file pbio.3003557.s011.docx]

Table S7: Summary of coefficient of variation (CV) values for P*_sidA_yfp* and P*_uvrA_yfp* promoter fusions in wild type (+/- MMC damage) and *ΔlexA* background.

| background | *ΔlexA* | *ΔlexA* | *wild type* | *wild type* | *wild type* | *wild type* |
| --- | --- | --- | --- | --- | --- | --- |
| Promoter fusion | P*_sidA_YFP* | P*_uvrA_YFP* | P*_sidA_YFP* | P*_sidA_YFP* | P*_uvrA_YFP* | P*_uvrA_YFP* |
| DNA damage | - | *-* | *-* | *+* | *-* | *+* |
| Standard deviation | 0.099228 | 0.00742649 | 0.00265565 | 0.00135103 | 0.03245838 | 0.00639609 |
| mean | 0.109759 | 0.005714 | 0.00348718 | 0.00256387 | 0.059028 | 0.00838 |
| CV | 0.9040534 | 1.29970047 | 0.7615447 | 0.52695137 | 0.54988103 | 0.76325687 |

Data for the *ΔlexA* strains are derived from the experiment represented in Fig. 4E. For the wild type strains, data for the no damage control were pooled from experiments represented in Fig. 2C, Fig. S2D and Fig. S2E. Data for promoter fusions in wild type background exposed to DNA damage were derived from experiment represented in Fig. 2C.
